# Evaluation of Leaf Mineral, Flavonoid, and Total Phenolic Content in Spider Plant Germplasm

**DOI:** 10.3390/molecules26123600

**Published:** 2021-06-11

**Authors:** Fhatuwani Thovhogi, Godwin Richard Ainamensa Mchau, Eastonce Tendayi Gwata, Nombasa Ntushelo

**Affiliations:** 1 Department of Horticulture, School of Agriculture, University of Venda, Private Bag X5050, Thohoyandou 0950, South Africa; ainamensa.mchau@univen.ac.za; 2 Department of Crop Science, School of Agriculture, University of Venda, Private Bag X5050, Thohoyandou 0950, South Africa; ectgwata@gmail.com; 3 Agricultural Research Council (Infruitec-Nietvoorbij), Private Bag X5026, Stellenbosch 7599, South Africa; NtusheloN@arc.agric.za

**Keywords:** flavonoid, genotype, minerals, phenolic compounds, spider plant

## Abstract

Spider plant (*Cleome gynandra* L.) is an important leafy vegetable that grows naturally in many parts of the world. The leaves are highly nutritious and are used mainly for human consumption. The mineral content and phenolic compounds of 17 genotypes (local and exotic) of spider plant and four standards (swiss chard, jute mallow, cowpea, and pumpkin) were investigated. Leaf samples were harvested from plants raised at Thohoyandou, South Africa. Exotic genotypes were superior to local genotypes for most of the minerals. Swiss chard possessed significantly high levels of some minerals such as iron and manganese in comparison with exotic spider plant genotypes. The calcium content in the local (‘MP-B-3-CG’) and exotic (‘GPS’) genotypes was >30.0% and >60.0% higher than in swiss chard, respectively. Total phenolics among spider plant genotypes ranged from 9.86 to 12.21 mg GAE/g DW and were superior to pumpkin. In addition, the spider plant genotypes varied significantly in the antioxidant capacity as estimated by the 2,2 diphenyl-1-picrylhydrazyl method and ferric-reducing antioxidant power. The main flavonoid in the leaves of spider plant genotypes was quercetin-3-rutinoside. Crotonoside (glycoside) was detected in all the spider plant genotypes and swiss chard. A positive correlation was observed between total phenolic content and each of the three flavonoids. The PCA biplot associated exotic genotypes (‘ML-SF-29′, ‘PS’, ‘TZ-1’, and ‘GPS’) and local genotypes (‘ML-3-KK’, ‘ML-13-SDM’, and ‘ML-12-TMP’) with high Al, Fe, Zn, N, and TPC. Cluster analysis indicated high “distant groups” between exotic and local genotypes of spider plant. These results indicated that some of the local germplasm of spider plant was largely inferior to the exotic germplasm in terms of their mineral composition but contained considerable quantities of quercetin-3-rutinoside, particularly in the local genotypes ‘MP-B-2-CG’ and ‘MP-B-1-CG’. There is a need for genetic improvement of the local germplasm in some of the minerals particularly to benefit the end-users.

## 1. Introduction

Spider plant (*Cleome gynandra* L.) is an important indigenous leafy vegetable that is important particularly in the diet of many rural communities in Africa, including South Africa [1]. It belongs to the family *Cleomaceae* and it is widely distributed as a weed in tropical and subtropical regions of the world, including South Africa [2]. This vegetable can play a vital role in food security and income generation particularly for marginalized rural communities in Africa. The vegetable is collected from the wild during the rainy season in many parts of the world [3,4]. However, in parts of East Africa, the vegetable is cultivated by smallholder growers [5]. Compared to other leafy vegetables, it is highly nutritious and is classified as a functional food due to the presence of phenolic compounds that are beneficial for human health [6].

In Africa, the consumption of indigenous leafy vegetables such as spider plant depends on a variety of factors such as poverty status and degree of urbanization [4]. In general, the leafy indigenous vegetables are an inexpensive source of dietary minerals, trace elements, and antioxidant phytochemicals [6]. The young tender leaves that are preferred by end-users are boiled and consumed as a side dish or dried for consumption during the off-season [7]. Indigenous leafy vegetables are also rich in flavonoids, which have anti-inflammatory, antibacterial, antihistaminic, and antimutagenic properties. They also possess micro- and macro-elements.

Micronutrient deficiency is a huge problem that affects the health of many people worldwide [8]. This is mainly due to changes in dietary preferences resulting from social economic development [9]. In many African countries, there is a decline in the consumption of indigenous leafy vegetables such as the spider plant in exchange for exotic vegetables [10]. Consumption of spider plant can be an alternative means to improve human health since the vegetable is rich in minerals such as, iron (Fe), manganese (Mn), and zinc (Zn).

The variation in the nutritional and phytochemical content of plant foods is influenced by a wide range of factors such as geographical location, season, cultivar, and physiological state [11,12]. A study conducted in South Africa showed variation in the nutritional content of spider plant across two provinces due to geographical and climatic factors [6]. Conversely, a similar study carried out in Malawi showed no significant differences in protein, fiber, and vitamin C content of the vegetables [7]. However, limited research has been conducted in South Africa on the nutritional composition of spider plant. Moreover, the studies utilized samples that were collected from the wild or purchased from the market [6,13,14] or planted in the field [15]. However, for those planted in the field, chicken and cattle manure were added [15]. There is merit in evaluating the nutrient composition of this vegetable cultivated in fertilizer-free environments. Most of the studies conducted in South Africa concentrated largely on the mineral, vitamin, and total phenolics profiling of spider plant [6,13,14,15].

The spider plant is also rich in secondary metabolites such as glucosinolates and flavonoids [11,16]. Glucosinolates are the sulfur and nitrogen-containing secondary metabolites responsible for the bitter taste in cruciferous vegetables. Genetic and environmental factors contribute to the variation in the amount and pattern of glucosinolates [17]. Flavonoids are the largest and most abundant group of secondary metabolites with marked antioxidant properties [18]. Some of the properties of flavonoids include free radical scavenging, strong antioxidant activities in preventing oxidation, inhibition of low lipoproteins, inhibition of hydrolytic and oxidative enzymes, and anti-inflammatory actions [19]. The determination of the radical scavenging activity of the spider plant using the 2,2 diphenyl-1-picrylhydrazyl (DPPH) free radical scavenging activity method is dependent on concentration [13] and time [20]. The leaves of the spider plant also contain phenolic compounds and other phytochemicals that have health beneficial properties [21]. Plant phenolics include phenolic acids, flavonoids, and condensed tannins.

Limited information is available on the quantification and identification of flavonoids of spider plant in South Africa. In addition, there is inadequate information regarding the variation in the mineral and phenolic content in spider plant in South Africa. Few studies utilized germplasm from different ecological regions in Kenya [16] and in the Netherlands [22]. Glucosinolates including glucocapparin, glucobrassicin, and 3-hydroxypropylglucosinolate as well as quercetin-3-O-rutinoside and several hydroxycinnamic acid derivatives were reported [11,16,22]. The content and distribution of flavonoids among 91 edible plant species including spider plant were evaluated [23]. Also, plant secondary metabolites in six selected species (African nightshade, spider plant, amaranth, cowpea, common kale, and Ethiopian kale) from East Africa were identified and quantified [11]. The major flavonoids identified in the leaves and shoots of spider plant include quercetin, kaempferol, and isorhamnetin [11,16,22,23]. The objective of this study was to determine the nutritional composition in a wide range of spider plant genotypes that were collected from different agro-ecological zones in South Africa and exotic accessions from different countries as a prerequisite for their subsequent selection and genetic improvement. The study also identified and quantified the flavonoids in the leaves of the germplasm.

## 2. Results

### 2.1. Leaf Mineral Content

There were highly significant differences (*p* ≤ 0.001) among the genotypes from different ecological regions for most of the mineral contents including Mg, P, Al, Cu, Fe, Mn, and Zn (Table 1). The Fe content was six-fold higher than the Zn leaf content. The mean for the potentially toxic element Al was 202.72 mg/kg, while Fe and Mn attained 330.00 mg/kg DW and 200.50 mg/kg DW, respectively. Similarly, the leaf Ca content (14.6 g/kg DW) was more than double the leaf content of both Mg and P (Table 1). The exotic genotype ‘GPS’ contained the highest (6.5 g/kg DW) leaf Mg content followed by ‘PS’ (6.3 g/kg DW), ‘UG-SF-15′ (6.1 g/kg DW), and ‘IP-3′ (6.1 g/kg DW), which were comparable to local genotypes ‘ML-2-DD’ (6.4 g/kg DW), ‘ML-13-SDM’ (6.3 g/kg DW), and ‘ML-12-TMP’ (6.1 g/kg DW), but this was significantly lower than that observed for the standard leafy vegetable swiss chard (11.5 g/kg DW). In addition, the genotype ‘ML-13-SDM’ showed significantly high Cu content (15.70 mg/kg DW) and Fe (346.67 mg/kg DW), but these were similar to both pumpkin and swiss chard (Table 1). The local genotype ‘ML-2-DD’ attained the highest Al content (316.33 mg/kg DW), which was more than double the content in both pumpkin and jute mallow leaves.

The leaf Fe content in the leaves of exotic genotype ‘ML-SF-29’ (431.33 mg/kg DW) was higher than that observed in cowpea (54.28%), jute mallow (45.32%) and pumpkin (20.63%) (Table 2). The highest Mn leaf content (509.33 mg/kg DW) was attained by the exotic genotype ‘ML-SF-29’, which was >45.0% higher than in the leaves of cowpea (48.10%), jute mallow (46.33%), and pumpkin (75.85%) (Table 2). Similarly, the leaf Zn content (75.00 mg/kg DW) in the exotic genotype ‘UG-SF-15’ was >50.0% higher than in both cowpea and jute mallow (Table 2). The leaf Fe content in the local genotype ‘ML-14-MAG’ (419.33 mg/kg DW) was similar to the leaf Fe content in the standard genotype swiss chard (405.67 mg/kg DW). However, the leaf Fe content in the local genotype was at least seven-fold higher than that observed for the rest of the standard genotypes (Table 2). In comparison with the highest leaf Mn content of the genotype ‘ML-2-DD’ (302.33 mg/kg DW), swiss chard was approximately 44% higher. Similarly, the Zn leaf content in the genotype ‘ML-13-SDM’ (63.67 mg/kg DW) was 58% higher than that observed in the standard genotype cowpea (41.37%) and jute mallow (41.89%) (Table 2).

The mineral content was generally higher in the genotypes from Limpopo province in comparison with the Mpumalanga genotypes (Table 1). However, some of the genotypes showed similar mineral profiles. For, instance the leaf Fe content from genotypes collected in Limpopo province ranged from 235.00 to 419.33 mg/kg DW, while in Mpumalanga, it ranged from 289.00 to 359.67 mg/kg DW. These genotypes ‘ML-14-MAG’, ‘ML-2-DD’, ‘ML-12-TMP’, and ‘ML-3-KK’ from Limpopo had high leaf Fe content greater than 359.67 mg/kg DW, which was higher when compared with all the genotypes from Mpumalanga Province. This trend was also observed with the leaf Al, Cu, and Mn content. The leaf zinc content was similar between genotypes from Limpopo and Mpumalanga province, but ‘ML-13-SDM’ (63.67 mg/kg DW) and ‘MP-B-1 CG’ (59.00 mg/kg DW) had high Zn leaf content when compared to other local genotypes.

### 2.2. Polyphenol Content and Antioxidant Activity

There were highly significant (*p* ≤ 0.001) differences among the spider plant genotypes in both polyphenol content and antioxidant activity. The highest (12.21 mg GAE/g) and lowest (9.86 mg GAE/g) total phenolic content (TPC) were attained by ‘ML-13-SDM’ and ‘ML-SF-29)’, respectively (Table 3). The highest antioxidant activity as measured by the DPPH free radical scavenging activity was observed for the local spider plant genotype ‘ML-14-MAG’. This antioxidant activity was significantly higher (*p* < 0.05) than in each of the standard common leafy vegetables except in cowpea. In addition, the antioxidant activity determined by the ferric-reducing antioxidant power (FRAP) in all the five exotic spider plant genotypes was significantly (*p* < 0.05) lower than in the local genotype (‘ML-6-BTK’).

When comparing spider plant genotypes from different ecological regions in South Africa, the genotypes from different agro-ecological zones in Limpopo and Mpumalanga did not differ significantly in total phenolic content. Meanwhile, a significant difference was found for antioxidant activity as measured by the DPPH method between genotype from Limpopo and Mpumalanga province with most of the genotypes from Limpopo attaining high DPPH of 22.68%, 22.55%, and 21.98% for ‘ML-14-MAG’, ‘ML-5-TGM’, and ‘ML-6-BTK’, respectively when compared to 15.14% for ‘MP-B-1-CG’, which was the highest for genotypes from Mpumalanga Province (Table 3).

### 2.3. Phenolic Compounds

Two local genotypes namely ‘MP-B-2-CG’ and ‘MP-B-1-CG’ attained significantly (*p* < 0.05) higher quercetin (QC) than all the exotic genotypes (Table 4). The highest QC content (1.76 × 10^−2^ mg/g) among the exotic genotypes was only 46.31% of the best local ‘MP-B-2-CG’ genotype (Table 4). Similarly, the local genotypes were superior to the exotic genotypes as well as the standards in terms of quercetin-3-rutinoside (Q3R). Quercetin 3-glucoside was identified in all the indigenous leafy vegetables in this study except in swiss chard (Table 4). The concentration of this flavonoid differed among the indigenous leafy vegetables. However, all the spider plant genotypes were inferior to jute mallow (7.34 × 10^−2^ mg/g) in terms of quercetin-3-β-d-glucoside (Q3βDG)

**Figure 1:**
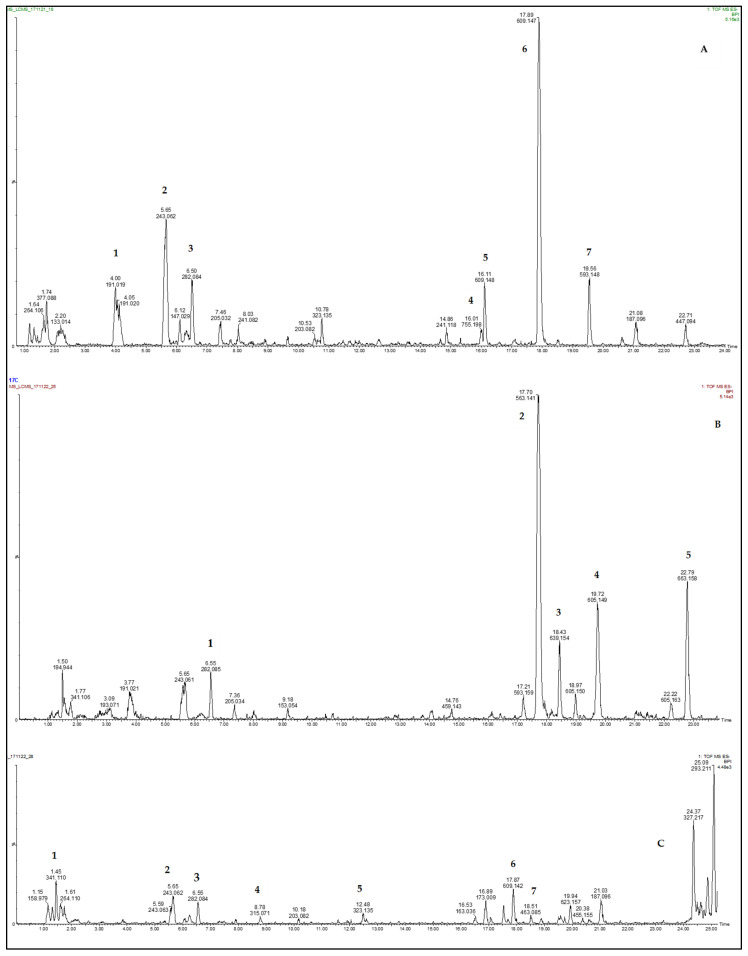

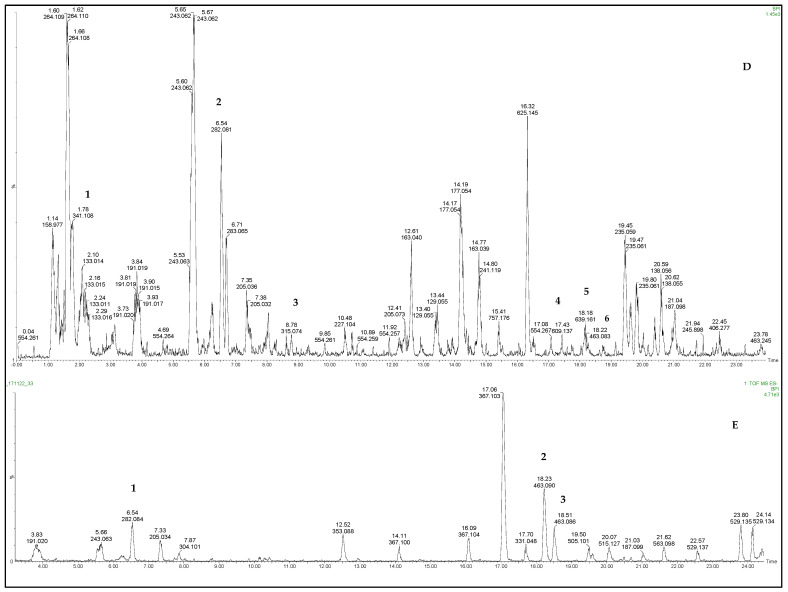
Chromatograms of spider plant (**A**), swiss chard (**B**), pumpkin (**C**), cowpea (**D**), and jute mallow (**E**).

The quercetin glycosides were present in spider plant, cowpea, jute mallow, and pumpkin leaves (Figure 1 and Table 5). Quercetin, quercetin 3-glucoside, and kaempferol 3-O-rutinoside were identified in the leaves of spider plant. In addition, crotonoside was identified in the leaves of spider plant and swiss chard (Figure 1 and Table 5). The flavonoids apiin and apigenin 7-6″-malonyl neohesperidoside were observed in swiss chard leaves. Furthermore, crotonoside was identified in the leaves of spider plant and swiss chard, but it was not detected in jute mallow, cowpea, and pumpkin (Figure 1 and Table 5).

### 2.4. Correlations among Mineral, Flavonoid Content, and Antioxidant Activity

A highly significant (*p* ≤ 0.01) positive correlation was observed between N and Ca (Table 6). Mg showed a positive significant (*p* < 0.05) correlation with each of P, Cu, N, Zn, and Fe and highly significant (*p* ≤ 0.01) correlation with Ca. QC also showed a highly significant (*p* ≤ 0.01) correlation with quercetin-3-rutinoside (Q3R) and Q3βDG. Weak positive correlations were observed between QC and FRAP (r = 0.37) and TFC (r = 0.33). However, both the total flavonoid content (TFC) and the antioxidant activity showed no significant correlation with any of the minerals (Table 6). Nonetheless, FRAP showed a positive significant (*p* < 0.01) correlation with DPPH and TFC.

### 2.5. Principal Component Analysis (PCA)

The first two principal components contributed 63.15% of the total variance in the data. The first principal component (F1) explained 35.06% followed by the second principal component (F2) with 28.09%. Variables that contributed the most to (F1) were P (0.92), N (0.87), TFC (0.79), Ca (0.72), Zn (0.70), and TPC (0.65). The first principal component increased with increasing P, N, TFC, Ca, Zn, and TPC and this suggests that these variables vary together. Thus, if one of these variable increased, then the remaining ones tended to increase as well (Table 7). Furthermore, it was observed that F1 correlated most strongly with P. It would follow that genotypes with high values for the variables mentioned above tend to be high in P, N, TFC, Ca, Zn, and TPC, while genotypes with small values would have low amount of these nutrients. The second principal component (F2) was strongly correlated with five of the variables and increased with increasing Mg, Al, Cu, and Fe. This suggested that genotypes with high Mg tended to have high Al, Cu, and Fe (Table 7). Furthermore, Mg, Al, Cu, and Fe were positively correlated but negatively correlated to DPPH. Thus, Mg, Al, Cu, and Fe negatively correlated with DPPH, indicating that genotypes that were high in Mg, Al, Cu, and Fe tended to have low DPPH values.

**Figure 2:**
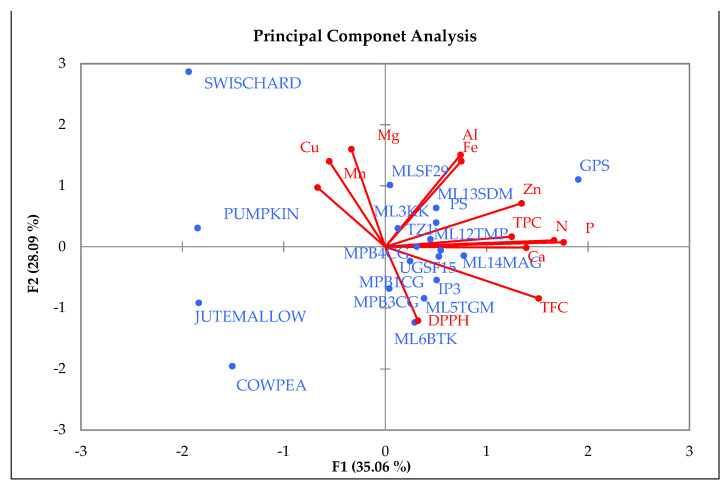
Principal component analysis of spider plant genotypes with mineral content, total polyphenol, and antioxidant activity.

The PCA biplot illustrated the relationship between the genotypes and 12 variables for mineral content, total polyphenol, and antioxidant activity. The variables on the first and second quadrant were positively correlated and included Al, Fe, Zn, N, Ca, TPC, TFC, and DPPH, and these variables were associated with all the spider plant genotypes. Swiss chard, jute mallow, cowpea, and pumpkin on the third and fourth quadrant were associated with Mg, Mn, and Cu (Figure 2). Four exotic genotypes (‘ML-SF-29’, ‘PS’, ‘TZ-1’, and ‘GPS’) and local genotypes (‘ML-3-KK’, ‘ML-13-SDM’, and ‘ML-12-TMP’) were associated with high Al, Fe, Zn, N, and TPC and low Ca, TFC, and DPPH. In addition, two exotic genotypes (‘UG-SF-15’ and ‘IP-3’) and six local genotypes (‘MP-B-4-CG’, ‘MP-B-3-CG’, ‘MP-B-1-CG’, ‘ML-5-TGM’, ‘ML-6-BTK’, and ‘ML-14-MAG’) were associated with high Ca, TFC, and DPPH while low on Al, Fe, Zn, N, and TPC.

### 2.6. Cluster Analysis

**Figure 3:**
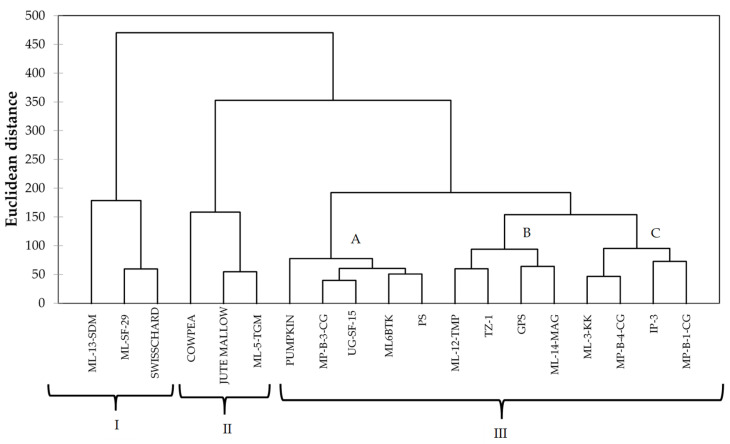
Complete linkage dendrogram showing existing groups in 15 spider plant accessions and four standards based on 12 variables for mineral content, total polyphenol count, and antioxidant activity.

The dendrogram produced five clusters out of 15 spider plant genotypes and four standards (Figure 3). Cluster I consisted of two spider plant genotypes and a standard. The exotic genotype ‘ML-SF-29’ was clustered with swiss chard, while the local genotype ‘ML-13-SDM’ was a singleton. A singleton is an accession that is placed separately from the rest of the genotypes in a cluster (Table 8). This type of genotype is more diverse and superior over other genotypes. The second cluster had two standards (cowpea and jute mallow) and local genotype ‘ML-5-TGM’. The third cluster consisted of 12 spider plant genotypes and one standard and was subdivided into sub-cluster A, which contained pumpkin as a singleton and four spider plant genotypes. The local genotypes ‘MP-B-3-CG’ and ‘ML-6-BTK’ were similar to exotic genotypes ‘UG-SF-15’and ‘PS’, respectively. Sub-cluster B contained two local genotypes from Limpopo ‘ML-12-TMP’and ‘ML-14-MAG’, which were similar to exotic genotypes TZ-1 and ‘GPS’, respectively (Figure 3 and Table 8).

## 3. Discussion

The results that were reported showed strong evidence that the leaves of spider plant contained a wide range of minerals that are useful in the human diet. The concentration of both micro- and macro-elements in the leaf tissue of the vegetable was generally comparable with those reported previously in similar studies [13,16,24]. Nonetheless, where differences exist in some individual minerals, it is likely due to the environmental factors such as edaphic attributes and management of the cropping systems. In the present study, the spider plants were raised without applying any fertilizers. In contrast, chemical [25] and organic [16] fertilizers were utilized in similar studies involving spider plant. Previous reports observed that the application of calcium ammonium nitrate fertilizers enhanced leaf calcium content and farmyard manure decreased Fe content but did not influence Zn or K content in spider plant [26]. In addition, the difference in agro-ecological regions between this study and similar investigations probably contributed to some of the individual differences in individual mineral levels. There is a considerable degree of diversity and environmental variations in Sub-Saharan Africa particularly in soil moisture availability, temperatures, and cropping systems [27].

Variation in mineral concentration between spider plant germplasm suggested that there is potential for the genetic improvement of the local germplasm in terms of leaf mineral content. Therefore, future genetic improvement activities aimed at enhancing the leaf mineral content in this vegetable will need to identify genotypes that are stable in mineral content in the target production areas. In addition, genotypes that were significantly higher in specific minerals could be utilized as source materials for introgressing such traits (also referred to as attributes or distinct plant characteristics) into the inferior genotypes. For instance, the two local genotypes (‘ML-13-SDM’ and ‘ML-12-TMP’) that were comparable to exotic genotypes ‘GPS’ and ’IP-3’ in TPC could be utilized in a breeding program aimed at enhancing this trait.

The leaf extract of the local spider plant genotypes (‘ML-6-BTK’, MP-B-1-CG’, and ‘MP-B-4-CG’) produced the highest FRAP values and were superior to both the exotic genotypes and all the standards. The DPPH and FRAP values in the leaf extract are an indication of the antioxidant potentials of the local and exotic spider plant genotypes showing that some of the local genotypes were superior in antioxidant properties. Therefore, the high antioxidant activities and ferric-reducing antioxidant power of the leaf extracts strongly supported the utilization of these vegetables. The relatively low antioxidant activity as measured by DPPH may be due to the difference in the concentration or dosage of the extract used. Previous studies reported that an increase in radical scavenging activity was dose or concentration dependent [13].

This study observed that quercetin glycosides were present in spider plant and the three standards (cowpea, jute mallow, and pumpkin) but differed in their concentrations. This agreed with the findings from previous studies [11,23]. The present study also showed that quercetin was the most abundant flavonoid in the spider plant leaf tissue, but the flavonoid content was generally high in the genotypes originating from South Africa (‘MP-B-2-CG’ and ‘ML-6-BTK’). Quercetin represents the main flavonoid in our daily diets among the polyphenols [28]. Quercetin is a versatile molecule with many pharmacological properties such as antioxidant, neurological, antiviral, anticancer, cardiovascular, and antimicrobial activities as well as the ability to protect the reproductive system [29,30,31,32]. Quercetin was found to have therapeutic potential for the treatment of breast cancer [33]. In a study involving male rats, quercetin in conjunction with sulfasalazine-induced alterations in steroidogenic enzyme activity, which enhanced organ weights, sperm integrity, and plasma hormone management, among other beneficial activities [34]. In addition, quercetin possessed antiviral activity during the early stage of infection by the influenza A virus [35]. The absence of detectable levels of phenolic compounds in swiss chard but their abundance in spider plant suggested that indigenous leafy vegetables such as the spider plant maybe superior to commercial leafy vegetables in some of the nutritional attributes. Therefore, the consumption of both indigenous and non-indigenous leafy vegetable types can provide a relatively wider spectrum of valuable nutrients that are necessary for human health. Nonetheless, the absence of some of the flavonoids (such as 3-hydroxypropyl glucosinolate and 4-methoxyglucobrassicin) that were detected in spider plant in other studies [11,22] could be attributed to the limited number of standards that were used in the present study. Furthermore, in a previous study, alteration of plants regulatory network which led to an accumulation of flavonoid, was attributed to UV-B radiation [36].

However, the presence of crotonoside (glycoside) in spider plant leaves was interesting in this study, since it is a potent tyrosine inhibitor with immunosuppressive effects on immune cells [37], antitumor activity [38], as well as selective inhibition in acute myeloid leukemia cells [39]. The study showed a positive relationship between quercetin and the derivatives (quercetin-3-rutinoside and quercetin-3-β-d-glucoside). The absence of a strong correlation between the antioxidant activity (using FRAP and DPPH) with the phenolic content agreed with the observations reported in a similar study involving the spider plant [40]. This suggests that the antioxidant activity is not entirely influenced by the phenolic compounds but other factors such as the presence of non-phenolic compounds. Antioxidant activity is partially influenced by other non-phenolic compounds such as ascorbates, reducing carbohydrates, tocopherols, carotenoids, and terpenes probably acting synergistically to produce the total antioxidant activity [41], and pigments as well as the synergistic effect among them could possibly contribute to the total antioxidant activity. The significant positive relationship between total phenolic content, quercetin, quercetin-3-β-d-glucoside, and quercetin-3-rutinoside could be useful in selection programs that are aimed at the concomitant improvement of both total phenolic content and flavonoids in spider plant. On the other hand, a positive relationship that was observed between Cu and Zn could be attributed partly to the role of Cu as a cofactor of the antioxidant enzyme Cu, Zn-superoxide [42].

The PCA biplot showed a high level of similarity between the spider plant genotypes and the four standards. All the spider plant accessions were clustered together in the first and second quadrant, while the standards (swiss chard, jute mallow, cowpea, and pumpkin) were isolated from the spider plant genotypes. This is an indication that the indigenous leafy vegetable spider plant is superior in terms of the nutritional composition when compared to the standards in this study. Four exotic genotypes and three local genotypes were associated with high Al, Fe, Zn, N, and TPC and low Ca, TFC, and DPPH and were positively correlated with each other. These genotypes could be used for spider plant genetic improvement. The cluster analysis showed the diversity between spider plant genotypes from different agro-ecological regions and the four common leafy vegetables that were used as standards in the study. The clustering pattern showed that some of the exotic genotypes were similar to the local genotypes. The clusters showed three groups consisting of local genotypes from Limpopo ‘ML-12-TMP’ and ‘ML-14- MAG’ clustering with exotic genotypes ‘TZ-1’ and ‘GPS’ and indicating a high level of similarity. In cases where clusters consisted of both local and exotic genotypes, it indicated that some of the local genotypes are comparable to exotic genotypes in terms of the nutritional composition.

## 4. Materials and Methods

### 4.1. Plant Material

Seventeen genotypes of spider plant (*Cleome gynandra* L.) were used in the study (Table 9). However, the total number of genotypes that were analyzed per specific attribute varied between 15 and 17 depending on the availability of sufficient leaf quantity for analysis. Four standard leafy vegetables that are common in many parts of Africa were included in the study. Three of these (namely cowpea, jute mallow, and pumpkin) were local traditional varieties, whereas the remainder (swiss chard) was a commercial variety.

### 4.2. Location and Planting

The experiments were conducted during the summer growing seasons (October–March). The plants were raised in the field at Thohoyandou, (22°95′ S; 30°48′ E, 595 m 437 a.s.l.). Daily temperatures at the location vary between 25 and 40 °C in summer and 18 and 26 °C in winter, and the rainfall is highly seasonal with 95% occurring between October and March. The annual average rainfall is about 500 mm. The soils at Thohoyandou are deep (>1500 mm), dystrophic, and well-drained clays with apedal structure, and they are classified as Hutton form [43]. The seed of the genotype was planted manually at a depth of 2.0 cm in a row of 2.0 m long and spaced at 30.0 cm within the row and 1.5 m between rows. Standard Cleome management practices were followed, and no chemical fertilization was applied. Leaves were harvested 6 weeks after germination.

### 4.3. Determination of Mineral Content

Leaf samples were harvested from five randomly selected plants per row and bulked. The leaf samples were washed with distilled water and dried to a constant weight overnight at 75 °C until there was no further moisture loss. The dried leaves were milled using a porcelain mortar and pestle and sieved through a 1 mm stainless steel sieve to obtain a homogenized sample. Approximately 5 g of the samples were weighed and stored in zip-sealed plastic bags at −20 °C until analysis. The milled samples (0.5 g) were ashed at 450 °C for 4 h. For digestion and filtration, a few drops of distilled water were added to the ashed contents, after which 2 mL of hydrochloric acid (HCl) was added. The samples were evaporated slowly to dryness in a water bath prior to the addition of 2.5 mL of freshly prepared 1:9 HCl solution to each sample. Then, the samples were filtered using Advantec 5B: 90 MM filter papers. The filtrate was diluted with de-ionized water at a ratio of 5:20. The diluted solution was analyzed for mineral elements using the Varian 720 Inductively Coupled Plasma Emission Spectrometer (ICP-OES, Frankfurt, Germany). The raw data from ICP-OES were taken for further calculations using the dry matter determined earlier as well as the weight of the weighed sample.

### 4.4. Preparation, Identification, and Quantification of Phenolic Compounds

Phenolic extracts were prepared by refluxing 2 g of the dried leaves samples in 20 mL of acidified methanol for 2 h at 60 ± 5 °C. The mixtures were centrifuged at 5000 rpm for 20 min, and the supernatants were separated and used for analysis of total phenolic content, total flavonoid content, and antioxidant activity. The identification and quantification of the phenolic content were determined using the LC-MS method [44]. Previous studies also utilized instruments with increased sensitivity and reliability to identify and quantify compounds [45]. The separation and identification of the phenolic compounds in the extracts were carried out using a Waters Synapt G2 quadrupole time-of-flight mass spectrometer (MS) (Milford, MA, USA). It was fitted with a Waters Ultra pressure liquid chromatograph (UPLC) and photo diode array (PDA) detector. The mass spectrometer was optimized for best sensitivity, the cone voltage was 15 V, the desolvation gas was nitrogen at 650 L/hr, and the desolvation temperature was 275 °C. Separation was achieved on a Waters HSS T3, 150 × 2.1 mm column. A gradient was applied using 0.1% (*v*/*v*) formic acid (solvent A) and acetonitrile containing 0.1% formic acid (solvent B). The gradient for the analysis of phenolic compounds started at 100% (solvent A) for 1 min and changed to 28% (solvent B) over 22 min in a linear way. Then, it went to 40% (solvent B) over 50 s and a wash step of 1.5 min at 100% (solvent B), followed by re-equilibration to initial conditions for 4 min. The flow rate was 0.3 mL/min and the column was kept at 55 °C. The injection volume was 2 µL. Rutin, citric acid, chlorogenic acid, and quercetin were used as standards. In a study involving flavonoid content analysis in safflower, rutin was also used as a standard [46].

### 4.5. Determination of Total Phenolic Content

The total phenolic content of the leaf extracts was determined according to the method by [47] with slight modifications. Briefly, 0.1 mL of acidified methanolic extract was mixed with 5 mL distilled water in a 50 mL volumetric flask. Folin–Ciocalteu’s reagent (2.5 mL) and 7.5 mL 15% sodium carbonate solution were added, mixed thoroughly, made up to 50 mL, and allowed to react for 30 min. The absorbance of the reaction mixture was read at 760 nm with a 96-well microplate spectrophotometer. The result was expressed as mg of gallic acid equivalent (GAE) per g of the sample.

### 4.6. Determination of Antioxidant Activity

The antioxidant properties of the plant extracts may be influenced by many factors such as the method used for extraction, the composition of the extract, and the type of procedure used [48]. In the present study, the antioxidant activity of the leaf extract was measured using the DPPH free radical scavenging activity and FRAP methods.

#### 4.6.1. 2,2 Diphenyl-1-picrylhydrazyl Free Radical Scavenging Activity (DPPH)

The DPPH radical scavenging activity was determined according to the method by [49] with slight modifications. An aliquot (10 µL) of acidified methanolic extract was mixed with distilled water (90 µL) and 3.9 mL of methanolic 0.1 mM DPPH solution. The mixture was thoroughly vortexed and kept in the dark for 30 min, and the absorbance was read at 515 nm. The result was expressed as the percentage inhibition of the DPPH radical. The percentage inhibition of the DPPH radical was calculated according to the following equation:

% Inhibition of DPPH = [Abs control − Abs sample/ Abs control] × 100(1)

where: Abs control is the absorbance of the DPPH solution without the extract.

#### 4.6.2. Ferric-Reducing Antioxidant Power

The reducing power assay was determined according to the method of [47] with slight modifications. Approximately, 100 µL of the extract was placed in a test tube, and the volume adjusted to 1 mL with methanol. Phosphate buffer (2.5 mL 0.2 M, pH 6.6) and 2.5 mL 1% potassium ferricyanide were added to the tube and vortexed. The mixture was left for 20 min at 50 °C, in a water bath. After incubation, 2.5 mL 10% (*w*/*v*) trichloroacetic acid was added, and the mixture centrifuged at 5000 rpm for 20 min; then, 2.5 mL of the supernatant was taken and mixed with 2.5 mL distilled water and 0.5 mL 0.1% (*w*/*v*) ferric chloride in a test tube, and the absorbance was measured at 700 nm. Higher absorbance indicates higher reducing power.

### 4.7. Statistical Analysis

Data obtained were subjected to analysis of variance (ANOVA) using SAS software (Version 9.4; SAS Institute Inc., Cary, NC, USA). The least significant difference test was used to separate means [50]. The mean values were statistically significant at *p* < 0.05. Pearson’s correlation coefficient test was used to measure the association between variables using SAS software (Version 9.4; SAS Institute Inc., Cary, NC, USA). Principal component analysis (PCA) was performed to determine the significant variables (mineral content, total phenols, and antioxidant activity) that contributed to the variation between spider plant genotypes from different agro-ecological regions and among local genotypes. Hierarchical cluster analysis based on the mineral content, total phenols, and antioxidant activity data were performed to generate a dendrogram describing the variability and similarity between genotypes. These data were subjected to cluster analysis, and the Euclidean measure of distance was used to estimate the genetic distance between genotypes.

## 5. Conclusions

The spider plant genotypes differed in the concentration of minerals, total phenolics, antioxidant capacity, and flavonoids. There were variations between local spider plant genotypes from different agro-ecological zones of South Africa for some of the nutritional composition; thus, these genotypes can be utilized in future genetic programmes. In addition, the bioavailability of the minerals that were assayed in this study could be useful to end-users. In future, there will be merit in the genetic improvement of the nutrient profile of the spider plant genotypes particularly where they were inferior to the exotic genotypes. Distant groups were observed for some of the local and exotic genotypes of spider plant, and this indicated potential for the genetic enhancement of this leafy vegetable.

## Figures and Tables

**Table 1:** Mineral content of exotic and local genotypes of spider plant and four standards (swiss chard, pumpkin, cowpea, and jute mallow).

g/kg DW					mg/kg DW				
Genotypes	N	Ca	Mg	P	Al	Cu	Fe	Mn	Zn
GPS (E)	67.3 ^a^	19.1 ^a^	6.5 ^b^	8.4 ^ab^	256.3 ^bc^	14.7 ^bcd^	409.3 ^ab^	123.3 ^fg^	71.3 ^ab^
UG-SF-15 (E)	66.7 ^ab^	15.6 ^bcd^	6.1 ^bcd^	6.8 ^bcde^	152.0 ^ghij^	15.4 ^bc^	275.7 ^def^	130.3 ^fg^	75.0 ^a^
PS (E)	65.6 ^ab^	15.5 ^bcd^	6.3 ^bc^	7.9 ^abc^	195.0 ^defgh^	14.5 ^bcd^	367.3 ^abcd^	109.7 ^fg^	65.3 ^bc^
ML-5-TGM (L)	64.4 ^ab^	14.8 ^bcde^	4.5 ^efg^	7.1 ^abcd^	146.7 ^ghij^	9.6 ^gh^	235.0 ^f^	199.3 ^bcdefg^	58.0 ^def^
ML-SF-29 (E)	63.7 ^ab^	16.3 ^abc^	5.5 ^cbde^	6.3 ^cde^	299.7 ^ab^	11.6 ^defgh^	431.3 ^a^	509.3 ^a^	50.3 ^ghij^
ML-3-KK (L)	62.6 ^abc^	13.7 ^bcdef^	4.9 ^cdefg^	7.3 ^abcd^	254.3 ^bc^	11.3 ^defgh^	373.3 ^abc^	169.3 ^defg^	52.0 ^efghij^
MP-B-3-CG (L)	61.4 ^abcd^	15.3 ^bcd^	4.7 ^defg^	6.8 ^bcde^	178.3 ^efghi^	11.6 ^defgh^	289.3 ^cdef^	134.0 ^fg^	47.0 ^ijk^
ML-13-SDM (L)	60.9 ^abcd^	15.3 ^bcd^	6.3 ^bc^	8.7 ^a^	201.3 ^cdefg^	15.7 ^abc^	346.7 ^abcde^	285.3 ^bc^	63.7 ^cd^
IP-3 (E)	60.5 ^abcd^	16.4 ^abc^	6.1 ^bcd^	6.2 ^de^	210.3 ^cdef^	10.2 ^fgh^	288.7 ^cdef^	169.3 ^defg^	52.0 ^efghij^
ML-14-MAG(L)	60.4 ^abcd^	16.6 ^abc^	5.2 ^bcdef^	7.1 ^abcd^	231.0 ^cde^	12.5 ^cdefg^	419.3 ^ab^	175.7 ^cdefg^	48.7 ^hij^
MP-B-1-CG (L)	60.1 ^abcd^	12.8 d^efg^	5.5 ^bcde^	7.3 ^abcd^	177.7 ^efghi^	12.3 ^cdefgh^	331.0 ^bcde^	217.0 ^bcdef^	59.0 ^cde^
MP-B-4-CG (L)	59.2 ^abcd^	14.6 ^bcde^	4.9 ^cdefg^	7.1 ^abcd^	246.3 ^bcd^	12.4 ^cdefg^	359.7 ^abcd^	157.3 ^efg^	55.3 ^efgh^
ML-12-TMP (L)	57.8 ^abcd^	15.7 ^bcde^	6.1 ^bcd^	7.8 ^abcd^	249.0 ^bcd^	13.8 ^bcde^	399.3 ^ab^	139.7 ^fg^	51.7 ^fghij^
Pumpkin (S)	55.8 ^abcd^	14.1 ^bcde^	6.1 ^bcd^	4.4 ^f^	135.7 ^ij^	16.3 ^ab^	342.3 ^abcde^	123.0 ^fg^	41.0 ^kl^
MP-B-2-CG (L)	55.8 ^abcd^	13.8 ^bcdef^	5.4 b^cde^	6.8 ^bcde^	219.7 ^cdef^	13.4 b^cdef^	352.0 ^abcd^	280.0 ^bcd^	54.0 ^efghi^
ML-2-DD (L)	54.8 ^bcde^	16.9 ^ab^	6.4 ^bc^	7.9 ^abcd^	316.3 ^a^	9.9 ^gh^	405.0 ^ab^	302.3 ^b^	50.3 ^ghij^
ML-6-BTK (L)	54.7 ^bcde^	13.4 ^cdef^	4.7 d^efg^	5.3 ^ef^	167.7 ^fghi^	8.9 ^h^	254.3 ^ef^	137.0 ^fg^	48.7 ^hij^
Cowpea (S)	51.2 ^cde^	15.2 ^bcd^	3.8 ^fg^	4.1 ^f^	124.3 ^ij^	10.7 ^efgh^	196.7 ^f^	264.3 ^bcde^	37.3 ^l^
Swiss chard (S)	50.0 ^de^	11.8 ^efg^	11.5 ^a^	3.9 f	256.3 ^bc^	18.9 ^a^	405.7 ^ab^	436.3 ^a^	56.0 ^efg^
Jute mallow (S)	49.7 ^de^	10.8 ^g^	3.7 ^fg^	4.3 ^f^	97.0 ^j^	14.1 ^bcd^	236.0 ^f^	273.3 ^bcd^	37.0 ^l^
TZ-1 (E)	4.37 ^e^	9.8 ^g^	3.7 ^g^	5.3 ^ef^	142.3 ^hij^	9.2 ^gh^	229.0 ^f^	91.0 g	45.7 ^jk^
Mean	58.4	14.5	5.6	6.5	202.7	12.7	330.0	200.5	53.3
R^2^ (%)	52.99	62.88	82.72	75.44	82.44	70.61	70.82	79.89	88.29
C.V. (%)	12.56	13.89	16.23	15.63	16.65	16.33	17.13	32.70	8.30
LSD	12.77	3.50	1.60	1.80	46.39	3.54	56.27	85.85	5.17

^a–l^ Means with the same superscript in a column are not significantly different (*p* < 0.05). E = exotic; L = local; S = standard; N = nitrogen; Mg = magnesium; Ca = calcium; P = phosphorus; Al = aluminum; Cu = copper; Fe = iron; Mn = manganese; and Zn = zinc.

**Table 2:** Percentage deficit between the highest (**a**) exotic and (**b**) local spider plant genotype and individual standard leafy vegetable.

**(a) Exotic highest spider plant genotype**			
**Genotype**	**Fe (ML-SF-29)**	**Mn (UG-SF-15)**	**Zn (ML-SF-29)**
Cowpea	54.28%	48.10%	50.22%
Jute mallow	45.32%	46.33%	50.66%
Pumpkin	20.63%	75.85%	45.33%
Swiss chard	5.90%	14.33%	25.33%
**(b) Local highest spider plant genotype**			
	**Fe (ML-14-MAG)**	**Mn (ML-2-DD)**	**Zn (ML-13-SDM)**
Cowpea	53.10%	12.57%	41.37%
Jute mallow	44.72%	9.59%	41.89%
Pumpkin	18.36%	59.32%	35.61%
Swiss chard	3.26%	−44.32%	12.05%

**Table 3:** Total polyphenol content and antioxidant activities of the spider plant genotypes, swiss chard, pumpkin, cowpea, and jute mallow.

Genotype	TPC (mg GAE/g)	DPPH (%)
ML-13-SDM (L)	12.21 ^a^	19.13 ^bcd^
ML-12-TMP (L)	12.15 ^a^	18.93 ^cd^
GPS (E)	12.24 ^a^	11.90 ^fgh^
IP-3 (E)	12.14 ^a^	13.16 ^efg^
MP-B-4-CG (L)	12.04 ^ab^	11.34 ^ghi^
TZ-1 (E)	12.04 ^ab^	8.70 ^hij^
MP-B-1-CG (L)	11.87 ^abc^	15.14 ^ef^
ML-5-TGM (L)	11.63 ^abcd^	22.55 ^ab^
UG-SF-15 (E)	11.45 ^abcde^	15.17 ^ef^
ML-14-MAG (L)	11.27 ^abcde^	22.68 ^a^
Swiss chard (S)	11.17 ^abcde^	5.06 ^k^
MP-B-3-CG (L)	10.76 ^bcdef^	11.59 ^ghi^
ML-3-KK (L)	10.76 ^bcdef^	5.37 ^jk^
Jute mallow (S)	10.62 ^cdef^	16.39 ^de^
ML-6-BTK (L)	10.38 ^def^	21.98 ^abc^
PS (E)	10.38 ^def^	8.07 ^ijk^
Cowpea (S)	10.27 ^ef^	22.55 ^ab^
ML-SF-29 (E)	9.86 ^f^	9.83 ^ghi^
Pumpkin (S)	7.46 ^g^	4.87 ^k^
Mean	11.01	13.92
R^2^ (%)	92.08	97.76
C.V. (%)	3.84	8.22
LSD	0.035	1.49

^a–k^ Means with the same superscript in a column are not significantly different (*p* < 0.05). E = exotic; L= local and S = standard; TPC = total phenolic content and DPPH = 2,2 diphenyl-1-picrylhydrazyl free radical scavenging activity.

**Table 4:** Phenolic compounds in spider plant genotypes, swiss chard, pumpkin, cowpea, and jute mallow.

Genotype	QC	Q3R	Q3βDG
	(×10^2^ mg/g)		
MP-B-2-CG (L)	3.80 ^a^	85.57 ^ab^	5.80 ^b^
MP-B-1-CG (L)	3.10 ^ab^	92.64 ^a^	4.45 ^bc^
ML-6-BTK (L)	2.17 ^bc^	75.49 ^bc^	3.69 ^c^
IP-3 (E)	1.76 ^cd^	65.34 ^cd^	4.53 ^c^
ML-2-DD (L)	1.71 ^cde^	66.00 ^cd^	1.93 ^de^
UG-SF-15 (E)	1.68 ^cdef^	61.45 ^def^	5.44 ^b^
ML-3-KK (L)	1.48 ^cdef^	60.25 ^def^	2.17 ^d^
MP-B-3-CG (L)	1.22 ^cdefg^	52.38 ^ef^	1.46 ^def^
ML-13-SDM (L)	0.92 ^defg^	63.57 ^cde^	0.76 ^efg^
PS (E)	0.90 ^defg^	63.85 ^cde^	1.80 ^def^
GPS (E)	0.85 ^defg^	63.88 ^cde^	1.65 ^def^
ML-12-TMP (L)	0.53 ^defg^	58.81 ^def^	0.00 ^g^
TZ-1 (E)	0.49 ^efg^	49.46 ^f^	0.61 ^efg^
MP-B-4-CG (L)	0.47 ^efg^	30.56 ^g^	0.45 ^fg^
ML-SF-29 (E)	0.45 ^gf^	61.03 ^def^	1.87 ^de^
Cowpea (S)	0.14 ^g^	0.00 ^i^	0.00 ^g^
ML-14-MAG (L)	0.00 ^g^	18.52 ^hg^	1.09 ^defg^
Pumpkin (S)	0.00 ^g^	9.8 ^hi^	1.60 ^def^
Swiss chard (S)	0.00 ^g^	0.00 ^i^	0.00 ^g^
Jute mallow (S)	0.00 ^g^	0.00 ^i^	7.34 ^a^
Mean	1.08 ± 0.06	48.93 ± 1.6	2.3 ± 0.12
R^2^ (%)	91.25	98.78	97.19
C.V. (%)	36.92	7.97	19.15

^a–i^ Means with the same superscript in a column are not significantly different (*p* < 0.05). E = exotic; L = local and S = standard; QC = Quercetin; Q3R = quercetin-3-rutinoside; Q3βDG = quercetin-3-β-d-glucoside.

**Table 5:** Molecules identified in spider plant and four standards (swiss chard, jute mallow, cowpea, and pumpkin).

Rank	RT	Fragmentations	Molecular Weight	Molecular Formula	Molecular Name
	(Min)	[M − H]^− (*m*/*z*)^	[M − H]^− (*m*/*z*)^		
	**Spider plant (*Cleome gynandra*)**				
1	3.87	111.0085	191.0184	C_6_H_8_O_7_	Citric acid
2	5.68	110.0231	243.0605	C_9_H_12_N_2_O_6_	Uridine
3	6.55	150.0441	282.0850	C_10_H_13_N_5_O_5_	Crotonoside
4	15.97	301.0343	755.2135	C_33_H_40_O_20_	Quercetin 3-(2G-rhamnosylrutinoside)
5	16.11	301.0392	609.1432	C_27_H_30_O_17_	Quercetin 3,4-di-*O*-glucoside
6	17.87	301.0392	609.1462	C_27_H_30_O_16_	Quercetin 3-*O*-rutinoside
7	18.50	300.0275; 301.0359	463.0868	C_21_H_20_O_12_	Quercetin 3′-glucoside
8	19.52	285.044	593.1566	C_27_H_30_0_15_	Kaempferol 3-*O*-rutinoside
9	24.19	151.0045	301.0371	C_15_H_10_0_7_	Quercetin
	**Swiss chard (*Beta vulgaris var. cicla*)**				
1	6.55	150.0418	282.0847	C_10_H_13_N_5_O_5_	Crotonoside
2	17.69	413.0872; 293.0449	563.1420	C_26_H_28_O_14_	Apiin
3	18.43	315.0501	639.1563	C_28_H_32_O_17_	Isorhamnetin-3,4’-diglucoside
4	19.74	455.0970; 293.0444	605.1505	C_28_H_30_O_15_	Vicenin-1 6’’-*O*-acetate
5	22.79	293.0457	663.1564	C_30_H_32_O_17_	Apigenin 7-6’’-malonyl neohesperidoside
	**Pumpkin (*Cucurbita pepo*)**				
1	1.45	203.0518; 185.0415	341.110	C_12_H_22_O_11_	Sucrose
2	5.65	-	243.062	C_14_H_12_O_4_	3-desmethyl 5-deshydroxy seleroin
3	6.55	168.0508; 140.0563	283.084	C_10_H_13_N_5_O_5_	8-hydroxy-2-deoxy guanosine
4	8.78	301.0396; 136.0171	315.071	C_16_H_12_O_17_	Quercetin 3-methyl ether
5	12.48	162.1292; 146.0570	323.135	C_19_H_20_N_2_O_3_	P-Hydroxyphenyl butazone
6	17.87	301.0392	609.142	C_27_H_30_O_16_	Rutin/quercetin-3-*O*-rutinoside
7	18.51	301.0371	463.085	C_21_H_20_O_12_	Isoquercetin/ Quercetin 3′-glucoside
	**Cowpea (*Vigna unguiculata*)**				
1	1.78	203.0518; 185.0415	341.108	C_12_H_22_O_11_	Sucrose
2	6.54	168.0508	282.081	C_10_H_13_N_5_0_5_	8-hydroxy-2-deoxy guanosine
3	8.78	301.0359	315.074	C_16_H_12_O_17_	Quercetin 3-methyl ether
4	17.43	300.0316	609.137	C_27_H_30_O_16_	Rutin/quercetin-3-*O*-rutinoside
5	18.18	329.1206	639.161	C_32_H_32_O_14_	Chartreusin
6	18.22	300.0291	463.083	C_21_H_20_O_12_	Isoquercetin/Quercetin 3′-glucoside
	**Jute mallow (*Corchorus olitorius*)**				
1	6.54	168.0508	282.084	C_10_H_13_N_5_O_5_	8-hydroxy-2-deoxy guanosine
2	18.23	300.0291	463.090	C_21_H_20_O_12_	Isoquercetin/Quercetin 3′-glucoside
3	1851	300.0291	463.086	C_21_H_20_O_12_	Isoquercetin/Quercetin 3′-glucoside

**Table 6:** Pearson correlation coefficient for mineral content, phenolic compounds, and antioxidant activities of spider plant.

	N	Ca	Mg	P	Al	Cu	Fe	Mn	Zn	QC	Q3R	Q3βDG	TPC	DPPH	FRAP	TFC
N	1.00															
Ca	0.72 **	1.00														
Mg	0.58 *	0.74 **	1.00													
P	0.53 *	0.57 *	0.69 *	1.00												
Al	0.29	0.56 *	0.45	0.39	1.00											
Cu	0.46 *	0.41	0.64 *	0.68 *	0.08	1.00										
Fe	0.41	0.60 *	0.56 *	0.58 *	0.87 **	0.43	1.00									
Mn	0.22	0.20	0.19	0.07	0.55 *	−0.03	0.45 *	1.00								
Zn	0.47 *	0.38	0.59 *	0.54 *	−0.17	0.74 **	0.05	−0.16	1.00							
QC	0.12	−0.26	0.06	−0.13	−0.22	−0.09	−0.29	0.10	0.08	1.00						
Q3R	0.12	−0.16	0.27	0.02	−0.14	0.01	−0.19	0.21	0.22	0.82 **	1.00					
Q3βDG	0.23	−0.09	0.14	−0.30	−0.30	−0.01	−0.35	0.08	0.26	0.84 **	0.64 *	1.00				
TPC	−0.22	0.01	0.17	0.32	−0.15	0.22	−0.13	−0.36	0.25	−0.02	−0.06	−0.13	1.00			
DPPH	0.18	0.14	0.03	0.04	−0.29	−0.03	−0.17	0.00	−0.01	0.06	−0.06	0.07	0.21	1.00		
FRAP	0.11	−0.05	−0.15	−0.08	−0.34	−0.11	−0.28	−0.14	0.02	0.37	0.03	0.20	0.26	0.62 *	1.00	
TFC	−0.13	−0.04	−0.14	−0.40	−0.25	−0.27	−0.39	−0.36	−0.03	0.33	−0.05	0.43 *	0.48 *	0.21	0.59 *	1.00

**, * = significant at the 1% and 5% probability level respectively. QC = quercetin; Q3R = quercetin-3-rutinoside; Q3βDG = quercetin-3-β-D-glucoside; TPC = total phenolic content; TFC = total flavonoid content; DPPH = 2,2-diphenyl-1-picrylhydrazyl free radical scavenging activity and FRAP = ferric-reducing antioxidant power assay.

**Table 7:** The principal component (PC) factor loadings and eigenvalues for mineral content, total polyphenol, and antioxidant activity.

Variables	F1	F2
N	0.867	0.056
Ca	0.723	−0.007
Mg	−0.174	0.833
Zn	0.700	0.371
Cu	−0.288	0.730
Mn	−0.348	0.507
Fe	0.390	0.730
P	0.915	0.039
Al	0.386	0.785
TPC	0.649	0.087
TFC	0.788	−0.440
DPPH	0.168	−0.629
Eigenvalue	4.207	3.371
Variability (%)	35.062	28.092
Cumulative %	35.062	63.154

**Table 8:** List of clusters of 15 spider plant genotypes and four standards (swiss chard, jute mallow, cowpea, and pumpkin) according to cluster analysis.

Cluster	Number of Genotypes	Genotypes	Type
I	3	ML-13-SDM	Local
		ML-SF-29	Exotic
		Swiss chard	Standard
II	3	Cowpea	Standard
		Jute mallow	Standard
		ML-5-TGM	Local
III	13	Pumpkin	Standard
		MP-B-3-CG	Local
		UG-SF-15	Exotic
		ML-6-BTK	Local
		PS	Exotic
		ML-12-TMP	Local
		TZ-1	Exotic
		GPS	Exotic
		ML-14-TMP	Local
		ML-3-KK	Local
		MP-B-4-CG	Local
		IP-3	Exotic
		MP-B-1-CG	Local

**Table 9:** Origins of the spider plant genotypes and other leafy vegetables that were used in this study.

Code/Name	Origin	Classification
1. ML-2-DD	Limpopo Province (South Africa)	Local
2. ML-3-KK	Limpopo Province (South Africa)	Local
3. ML-5-TGM	Limpopo Province (South Africa)	Local
4. ML-12-TMP	Limpopo Province (South Africa)	Local
5. ML-13-SDM	Limpopo Province (South Africa)	Local
6. ML-14-MAG	Limpopo Province (South Africa)	Local
7. ML-6-BTK	Limpopo Province (South Africa)	Local
8. MP-B-1-CG	Mpumalanga Province (South Africa)	Local
9. MP-B-2-CG	Mpumalanga Province (South Africa)	Local
10. MP-B-3-CG	Mpumalanga Province (South Africa)	Local
11. MP-B-4-CG	Mpumalanga Province (South Africa)	Local
12. TZ-1	Tanzania Local seed	Exotic
13. IP-3	Kenya (WorldVeg, Tanzania, Arusha)	Exotic
14. ML-SF-29	Malawi (WorldVeg, Tanzania, Arusha)	Exotic
15. PS	Tanzania (WorldVeg, Tanzania, Arusha)	Exotic
16. GPS	Tanzania (WorldVeg, Tanzania, Arusha)	Exotic
17. UG-SF-15	Uganda (WorldVeg, Tanzania, Arusha)	Exotic
18. Cowpea (*Vigna unguiculata*)	Limpopo Province (South Africa)	Local standard
19. Jute mallow (*Corchorus olitorius*)	Limpopo Province (South Africa)	Local standard
20. Pumpkin (*Cucurbita pepo*)	Limpopo Province (South Africa)	Local standard
21. Swiss chard (*Beta vulgaris var. cicla*)	Commercial variety	Local standard

Exotic genotype = genotypes originating in a foreign country. WorldVeg = World Vegetable Center.

## Data Availability

Not applicable.

## Sample Availability

Limited leaf samples are available from the authors.
